# The cathepsin S cysteine proteinase of the burrowing nematode *Radopholus similis* is essential for the reproduction and invasion

**DOI:** 10.1186/s13578-016-0107-5

**Published:** 2016-06-10

**Authors:** Ke Wang, Yu Li, Xin Huang, Dong-wei Wang, Chun-ling Xu, Hui Xie

**Affiliations:** Laboratory of Plant Nematology and Research Center of Nematodes of Plant Quarantine, Department of Plant Pathology, South China Agricultural University, Guangzhou, 510642 China; Department of Plant Pathology, Henan Agricultural University, Zhengzhou, 450002 China

**Keywords:** *Radopholus similis*, Cysteine proteinase, Cathepsin S, RNAi, Reproduction, Pathogenicity

## Abstract

**Background:**

The nematode *Radopholus similis* is an important migratory endoparasite of plants. Cysteine proteinases such as cathepsin S (CPS) play key roles during embryonic development, invasion, and pathogenesis in nematodes and many other animal parasites. This study was designed to investigate the molecular characterization and functions of a cathepsin S protease in *R. similis* and to find new targets for its control.

**Results:**

*Rs*-CPS of *R. similis*, *Hg*-CPS of *Heterodera glycines* and *Ha*-CPS of *H. avenae* are closely genetically related and share the same branch of the phylogenetic tree. *Rs*-*cps* is a multi-copy gene that is expressed in the esophageal glands, ovaries, testes, vas deferens, and eggs of *R. similis*. *Rs*-*cps* mRNA transcripts are expressed at varying levels during all developmental stages of *R. similis*. *Rs*-*cps* expression was highest in females. The neurostimulant octopamine did not significantly enhance the ingestion of the dsRNA soaking solution by *R. similis* but instead had a detrimental effect on nematode activity. The dsRNA soaking solution diffused into the body of *R. similis* not only through the esophageal lumen but also through the amphids, excretory duct, vagina, anus and cloacal orifice. We confirmed that RNAi significantly suppressed the expression level of *Rs*-*cps* and reproductive capability and pathogenicity of *R. similis*.

**Conclusions:**

Our results demonstrate that *Rs*-*cps* plays important roles in the reproduction, parasitism and pathogenesis of *R. similis* and could be used as a new potential target for controlling plant parasitic nematodes.

## Background

The burrowing nematode *Radopholus similis* is a migratory endoparasite of plants. *R. similis* is one of the most destructive plant pathogenic nematodes in the world and is listed as a quarantine pest in many countries and regions [1, 2]. *R. similis* has a wide host range and attacks more than 250 plant species [3]. *R. similis* severely harms banana, citrus, pepper, coffee and other agronomic and horticultural crops [4, 5] and is the most serious plant pathogenic nematode in most banana-growing areas of the world [6]. Despite extensive attention and research, controlling *R. similis* remains problematic worldwide, and effective approaches must be explored and established.

Proteolytic enzymes can be classified into four main groups: cysteine, serine, aspartyl and metallo proteinases. Cysteine proteinases are the most extensively studied [7]. Cysteine proteinases (EC 3.4.22) have been identified in a variety of organisms [8]. Most cysteine proteases are expressed and proteolytically active in the intestines, and these enzymes are the main digestive enzymes in nematodes and animal parasites [7]. Cysteine proteinases play important roles in embryogenesis and development, infection, parasitism, pathogenesis and immune evasion in nematodes and many other animal parasites [9–12]. Nematode cysteine proteinases mainly include cathepsin B-, L-, S-, K- and Z-like cysteine proteinases, and cathepsin L (CL) and cathepsin B (CB) have been extensively studied in recent years. CL is essential for embryogenesis and development in *Caenorhabditis elegans* [10, 13]. Guiliano et al. [14] demonstrated that CL proteinases in filarial nematodes are associated with larval molting and cuticle and eggshell remodeling. CB plays important roles in molting, the successful development of *Onchocerca volvulus* fourth-stage larvae [15] and in the invasion and pathogenesis of *Fasciola hepatica* and *Angiostrongylus cantonensis* [16, 17]. At present, CB genes have rarely been cloned in plant parasitic nematodes, and only CB of *Bursaphelenchus xylophilus* (GenBank No: GU130153) and *R. similis* (GU360972) are cloned. However, many CL genes have been cloned in plant parasitic nematodes, such as *Heteroderaavenae* (ACJ13100), *H. glycines* (Y09498), *H. schachtii* (ACJ13098), *Globodera virginiae* (ACJ13094), *G. Mexicana* (ACJ13096), *Meloidogyne incognita* (CAD89795), *Rotylenchulusreniformis* (AAY45870) and *B. xylophilus* (ACH56225) [7, 18, 19]. Li et al. [19, 20] reported that *Rs*-*cb*-*1* plays key roles in reproduction, development, hatching and pathogenesis in *R. similis.* However, the cathepsin S gene (*cps*) has rarely been reported, and only the *cps* genes of *H.glycines* [7], *R. similis* (EU659125) and *H. avenae* [21] have been cloned. The functions of *cps* in plant parasitic nematodes have not been explored. In this study, the expression and tissue localization of *Rs*-*cps* in *R. similis* were investigated using qPCR and in situ hybridization, and the roles of *Rs*-*cps* during reproduction and pathogenesis were studied using RNAi combinated with inoculation of carrot callus and tomato plants in pots. This study is the first to examine the functions of *cps* in plant parasitic nematodes and suggests a promising new target for controlling *R. similis*.

RNAi is a means by which dsRNA (double stranded RNA) induces sequence-specific posttranscriptional gene silencing [22]. This method was first developed in *C.elegans* and has subsequently been used in organisms ranging from lower fungi to higher mammals [22–26]. RNAi is also a very powerful tool for examining the functions of genes in plant nematodes and other organisms. For plant parasitic nematodes, in vitro RNAi (performed by soaking the nematodes in a solution of dsRNA in vitro) is the most widely used method, but the soaking time required to obtain optimal RNAi efficiency differs greatly among nematode species [19, 27–33]. The feeding mechanisms of plant parasitic nematodes vary. The infective second-stage juveniles of sedentary endoparasitic nematodes (such as cyst and root-knot nematodes) feed only following the establishment of a feeding site inside the root. Therefore, the primary barrier to successful in vitro RNAi is ensuring the ingestion of the dsRNA by the non-feeding second-stage juveniles of plant parasitic nematodes. In second-stage juveniles of *G. pallida*, *H. glycines* and *M. incognita*, dsRNA uptake can be induced by adding the neurotransmitter octopamine or resorcinol to successfully silence the targeted genes [27, 28]. In vitro RNAi-induced gene silencing has been achieved in *B. xylophilus* without exogenous neurotransmitter [31, 32]. The in vitro dsRNA soaking method has been used to induce RNAi to *R. similis* [19, 20, 34], but whether neurotransmitters facilitate the uptake of dsRNA by *R. similis* and affect nematode activity have not been examined. This study examined these questions using FITC as a visual marker.

## Methods

### Ethics statement

Animals were treated in strict accordance with the Animal Ethics Procedures and Guidelines of the People’s Republic of China. All animal procedures were approved by the Animal Ethics Committee of the South China Agricultural University.

### Nematode inoculum and plant growth conditions

*Radopholus similis* was collected from the roots of the ornamental plant *Anthurium andraeanum* and cultured in vitro on carrot disks at 25 °C [35]. At 50 d after inoculation, the cultured nematodes were extracted from the carrot disks according to the method described by Zhang et al. [34]. The tomato seeds used in this study were purchased from Guangzhou Changhe Seed Limited Company, Guangdong, and surface sterilized as described by Arshad et al. [36]. The sterilized seeds were sown in 1.5 L of sterilized soil and cultured in a 25 °C growth cabinet (16 h light/8 h dark photoperiods) for 30 d [20].

### RNA extraction, PCR amplification of *Rs*-*cps* and phylogenetic analysis

Total RNA was extracted from 20,000 mixed-stages nematodes of *R. similis* using TRIzol reagent (Invitrogen) and verified as previously described [34]. After being treated with RQ1 RNase-Free DNase (Promega) for 15 min at 37 °C, the cDNA was synthesized using the Reverse Transcriptase M-MLV (Takara). The full-length cDNA sequence of *Rs*-*cps* was amplified using the specific primers Rs-cps-S1 and Rs-cps-A1 (Table 1). The PCR product was purified, ligated into the vector pMD20-T (Takara) and transformed into *Escherichia coli* DH5α competent cells. Positive clones were confirmed by sequencing and the recombinant plasmid pMD20-Rs-cps was extracted for later use.

**Table 1:** Primers used in this study

Primer name	Sequence	Primer use
Rs-cps-S1	5′-AGTGCCCCTCCGAAATGT-3′	RT-PCR
Rs-cps-A1	5′-TGTCCGTTCTTCCGTTCA-3′	
SF	5′-TGTTGGCGGTCCCTGTG-3′	Southern blot
SR	5′-CGTGTTCGTGGACGGAGTT-3′	
Ish-T7S1^a^	5′-GGATCC*TAATACGACTCACTATAGGG*TGTTGGCGGTCCCTGTG-3′	ISH template
Ish-A1	5′- CCGTTCTCCTCGATGTAGTCA -3′	
Ish-S2	5′-TGTTGGCGGTCCCTGTG-3′	ISH template
Ish-T7A2^a^	5′-GGATCC*TAATACGACTCACTATAGGG*CCGTTCTCCTCGATGTAGTCA-3′	
qPCR-F	5′-AGAACTCCGTCCACGAACAC-3′	qPCR
qPCR-A	5′-GCCCACATTGCGCTTGCT-3′	
Actin-F	5′-GAAAGAGGGCCGGAAGAG-3′	qPCR
Actin-R	5′-AGATCGTCCGCGACATAAAG-3′	
CPS-T7S^a^	5′-GGATCC*TAATACGACTCACTATAGGG*TGTTGGCGGTCCCTGTG-3′	dsRNA template
CPS-A	5′- CGTGTTCGTGGACGGAGTT-3′	
CPS-S	5′-TGTTGGCGGTCCCTGTG-3′	dsRNA template
CPS-T7A^a^	5′-GGATCC*TAATACGACTCACTATAGGG* CGTGTTCGTGGACGGAGTT-3′	
eGFP-T7S^a^	5′-GGATCC*TAATACGACTCACTATAGGG*CAGTGCTTCAGCCGCTACC-3′	dsRNA template
eGFP-A	5′-AGTTCACCTTGATGCCGTTCTT-3′	
eGFP-S	5′-CAGTGCTTCAGCCGCTACC-3′	dsRNA template
eGFP-T7 A^a^	5′-GGATCC*TAATACGACTCACTATAGGG*AGTTCACCTTGATGCCGTTCTT-3′	

^a^The T7 promoter sequence is italiced

The amino acid sequences of the Rs-CPS protein and other CPS proteins were aligned using ClustalW. Based on the amino acid sequences of 25 CPS proteins from 17 species, a phylogenetic tree was constructed using the neighbor-joining method in MEGA 5.1 [37]. Bootstrap values were calculated from 1000 replicates.

### Southern blot hybridization

Approximately 10 μg of gDNA was obtained from *R. similis* and digested with *Nde*I and *EcoR*I. The digested DNA products were separated by 0.8 % (w/v) agarose gel electrophoresis and transferred to a Hybond N + membrane (Amersham) [38]. A 438-bp DIG-labeled probe was prepared using a PCR DIG Probe Synthesis Kit (Roche) with the specific primers SF and SR (Table 1). The membrane was hybridized for 18 h at 54.5 °C with the probe. Hybridization was performed using a Dig High Primer DNA Labeling and Detection Starter Kit I (Roche) according to the manufacturer’s instructions. After hybridization, the membrane was washed with 2 × SSC/0.1 % SDS for 15 min at 25 °C followed by 0.5 × SSC/0.1 % SDS for 30 min at 65 °C and examined. An equal amount of carrot callus gDNA was used as a control.

### In situ hybridization

In situ hybridization was performed as described by De Boer et al. [39] and Cheng et al. [40]. Specific sense (Ish-T7S1, Ish-A1) and antisense (Ish-S2, Ish-T7A2) primers (Table 1) were designed to amplify a 478-bp fragment based on the full-length sequence of *Rs*-*cps*. The purified PCR product served as the template to synthesize DIG-labeled sense and antisense RNA probes using DIG RNA labeling mix (Roche) according to the manufacturer’s instructions. Following fixation, the intact nematodes were cut into 2–5 fragments and hybridized with the DIG-labeled RNA probes (300 ng/mL). After hybridization, the stained nematode sections were examined and photographed using a 90i differential interference microscope (Nikon).

### Expression analysis of *Rs*-*cps* and qPCR

qPCR was used to detect the expression levels of *Rs*-*cps* in *R. similis* at different developmental stages. Total RNA samples were extracted from 100 *R. similis* females, males, eggs and juveniles using an RNeasy Micro kit (Qiagen), respectively. The extracted RNA was treated and quantified as previously described [34]. cDNA was synthesized using an iScript cDNA synthesis kit (Bio-Rad) according to the manufacturer’s instructions. Specific primers, qPCR-A and qPCR-S (Table 1), were designed according to the full-length sequence of *Rs*-*cps* to detect *Rs*-*cps* expression levels in *R. similis*. β-actin was amplified as a reference gene using the primers Actin-F/Actin-R (Table 1) [41]. qPCR was performed on a CFX-96 qPCR machine using iTaq Universal SYBR Green Supermix (Bio-Rad). The initial data analysis was performed using CFX-96 manager software, which created Ct values and extrapolated the relative levels of PCR products from standard curves. Melt curves were obtained routinely, which allowed the possibility of both contamination and primer dimers to be discounted [34, 38, 40]. All experiments were performed in triplicate with three biological replicates [20].

### Synthesis of *Rs*-*cps* dsRNA of *R. similis*

The specific primers CPS-T7S/CPS-A and CPS-S/CPS-T7A (Table 1) were designed to amplify a 438-bp fragment containing the T7 promoter. The purified PCR product was used to transcribe *Rs*-*cps* sense and antisense single-stranded RNA (ssRNA) using a ScriptMAXTM Thermo T7 Transcription Kit (TOYOBO). The corresponding dsRNA was synthesized and purified as described by Hannon [42]. The non-endogenous control dsRNA (the enhanced green fluorescent protein gene, e*gfp*) was synthesized using the specific primers eGFP-T7S/eGFP-A and eGFP-S/eGFP-T7 (Table 1).

### Effects of soaking *R. similis* with FITC and octopamine

The effects of FITC and octopamine on nematode activity and the effect of octopamine on dsRNA solution uptake by *R. similis* were assessed using FITC as a visual marker. The RNAi soaking method was performed as previously described [27]. Approximately 20,000 mixed-stage nematodes were collected from carrot disks and soaked in M9 buffer. The following compounds respectively were added to the above soaking solution at the indicated concentrations: (I) 0 (CK), 0.1, 0.2, 0.4, 0.8, 1.0 or 2.0 mg/mL fluorescein isothiocyanate (FITC) (Sigma-Aldrich) (stock made up at 20 mg/mL in DMF) [27]; (II) 0 (CK), 10, 25, 50, 75, 100 or 200 mM neurostimulant octopamine (Sigma-Aldrich); (III) FITC (0.8 mg/mL) or FITC(0.8 mg/mL) plus octopamine (50 mM). *Rs*-*cb*-*1* dsRNA was added to the soaking solution at a final concentration of 2.0 mg/mL [27]. Nematodes not treated with FITC or octopamine were used as controls. The nematodes were maintained in 1 mL of soaking solution with gentle agitation (100 rpm) in a dark rotary incubator at 25 °C for 4, 8, 12 and 24 h. After incubation, the nematodes were transferred to a 10-mL centrifuge tube and washed six times with sterile water to remove the soaking solution. FITC uptake was measured based on fluorescence intensity, and the effects of FITC and octopamine on nematode activity were detected using a fluorescence microscope (Nikon 90i) with appropriate filters. The nematodes were considered dead if they did not move after being pricked with a platinum wire [32]. For each treatment, 100 nematodes were effectively counted to quantify the effects of soaking *R. similis* with FITC and octopamine. Five biological replicates were performed.

### Effects of *Rs*-*cps* silencing on *R. similis*

Approximately 1000 mixed-stage nematodes were washed with DEPC water and then soaked in *Rs*-*cps* dsRNA solution (2.0 mg/ml) with gentle agitation in a dark rotary incubator (100 rpm, 25 °C) for 12, 24, 36 and 48 h, respectively. The treated nematodes were used in the following experiments. (I) Total RNA was extracted from 100 nematodes in each treatment group after they were washed with DEPC water, and qPCR was used to detect the silencing efficiency of *Rs*-*cps* in *R. similis*, as described above. These experiments were performed in triplicate with three biological replicates. (II) The phenotypic changes of nematodes were observed after soaking in *Rs*-*cps* dsRNA solution for 36 h. (III) A total of 30 female nematodes were inoculated onto carrot disks and cultured for 50 d at 25 °C, and then the reproductive rates (reproductive rate = final nematodes ⁄ initial nematodes) of the nematodes were calculated. Non-endogenous e*gfp* dsRNA treated nematodes (2.0 mg/mL) were used as a control. The soaking times for the controls were the same as those used for the *Rs*-*cps* dsRNAs. Untreated nematodes were used as a blank control (CK).

To detect the effect of *Rs*-*cps* silencing on the pathogenicity of *R. similis*, 1000 mixed-stage nematodes treated with *Rs*-*cps* dsRNA (2.0 mg/mL) for 36 h were inoculated onto each of the selected tomato plantlets. The selected plantlets were identical in height (approximately 20 cm) and growth conditions and were cultivated in a greenhouse as described above [20]. Nematodes treated with e*gfp* dsRNA for 36 h were used as the control. Untreated nematodes were used as a blank control. The plantlets were managed as usual except that they were not watered for the first 5 days [34]. After 60 days, the plant heights, fresh shoot weights and fresh root weights of the plants were measured and recorded. The symptoms of infected roots were photographed. The nematodes in the rhizosphere were isolated and quantified as described elsewhere [34, 43]. Five biological replicates were performed.

### Statistical analysis

All data in this study were analyzed using SAS 9.2 (SAS Institute, Cary, NC, USA) and subjected to one-way analysis of variance (ANOVA), and differences between treatments were compared using Duncan’s Multiple Range Test at *p* = 0.05.

## Results

### PCR amplification of *Rs*-*cps* and phylogenetic analysis

**Fig. 1:**
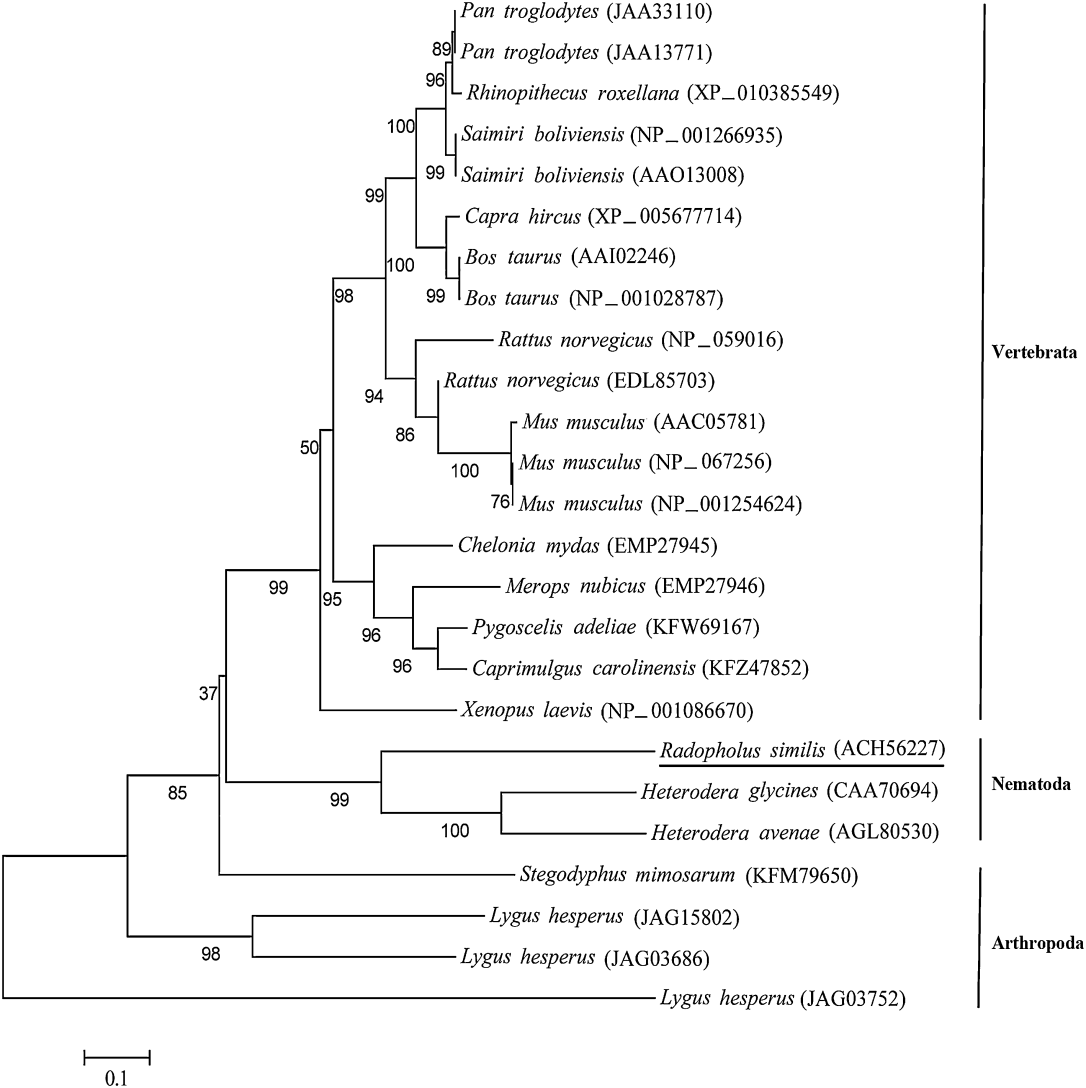
The phylogenetic relationships between *Radopholus similis* CPS and CPS proteins in other organisms. The phylogram was constructed based on amino acid sequences to describe the evolutionary relationships among 25 CPS proteins from 17 different species using MEGA 5.0. The numbers below the *branches* indicate the bootstrap values, which were calculated from 1000 replicates. The GenBank accession numbers of the sequences are in *brackets*. *Radopholus similis* CPS is *underlined*. *Distance scale* = 0.1

The 974-bp full-length cDNA sequence of *Rs*-*cps* was amplified using the specific primers Rs-cps-S1 and Rs-cps-A1 (Table 1) and confirmed by sequencing (result not shown). The sequencing results were consistent with the sequence in GenBank (GenBank: EU659125). The recombinant pMD20-Rs-cps plasmid, which includes the intact ORF (945 bp) of *Rs*-*cps*, was extracted for later use. The *Rs*-CPS protein encodes 314 aa with a theoretical molecular mass of 34.7 kDa. A 17 aa signal peptide with a cleavage site between Ala17 and Val18 was predicted by SignalP 3.0 at the N-terminus of the deduced *Rs*-CPS sequence. No transmembrane helix was predicted from the deduced *Rs*-CPS sequence using the TMHMM 2.0. The amino acid sequences alignment results revealed 56 and 45 % identity of *Rs*-CPS (ACH56227) with *Hg*-CPS from *H. glycines* (CAA70694) and *Ha*-CPS from *H. avenae* (AGL80530), respectively. A phylogenetic tree was generated using the neighbor-joining method based on the amino acid sequences of CPS proteins (Fig. 1). Twenty-five CPS proteins from 17 species were divided into three groups. *Rs*-CPS of *R. similis*, *Hg*-CPS of *H. glycines* and *Ha*-CPS of *H. avenae* were present in the same branch of the phylogenetic tree, within the Nematoda group, suggesting a close phylogenetic relationship. The remaining 22 CPS proteins were divided into two groups: Vertebrata and Arthropoda (Fig. 1).

### Southern blot hybridization

**Fig. 2:**
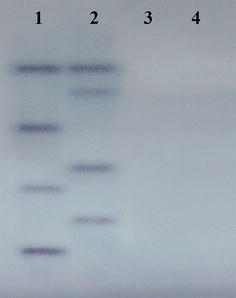
Southern blot analysis of *Rs*-*cps* in *Radopholus similis*. *Lanes* 1–2, gDNA from *R. similis* digested with *Nde*I and *EcoR*I; *lanes* 3–4, gDNA from carrot callus digested with *Nde*I and *EcoR*I. The digested genomic DNA was hybridized with a 438-bp DIG-labeled probe

To determine the gene copy number of *Rs*-*cps* in *R. similis*, *R. similis* gDNA was analyzed by Southern blot. The gDNA samples were digested with *Nde*I and *EcoR*I and then hybridized with a 438-bp DIG-labelled probe. Four strong hybridizing bands were observed in the *Nde*I- and *EcoR*I-digested *R. similis* gDNA samples. No hybridization signal was detected for digested carrot callus gDNA. Because there are no *Nde*I or *EcoR*I restriction sites in the genomic coding region or the cDNA sequence of *Rs*-*cps*, the results suggested that *Rs*-*cps* exists as a multi-copy gene in the *R. similis* genome (Fig. 2).

### Tissue localization and expression of *Rs*-*cps* mRNA *in R. similis*

**Fig. 3:**
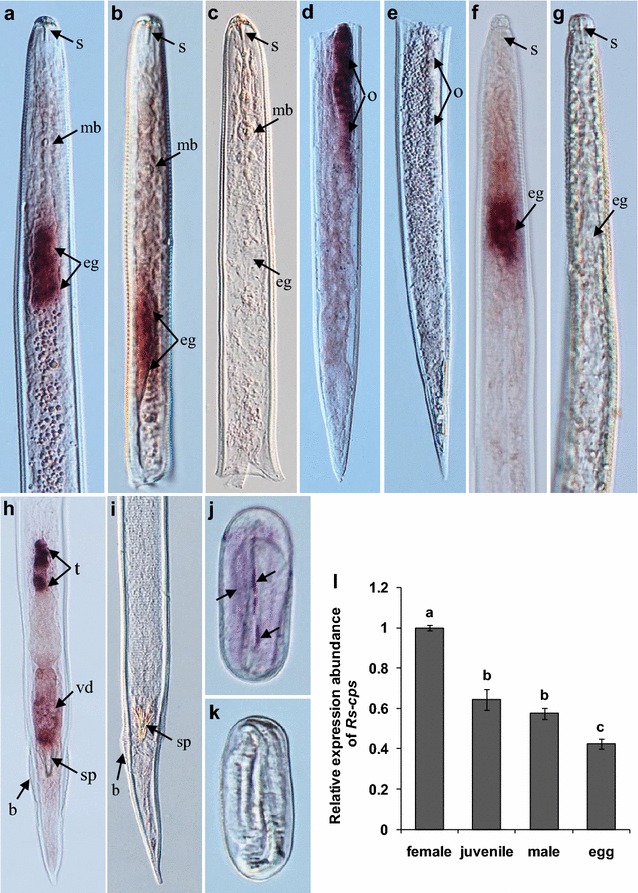
Tissue localization and expression of *Rs*-*cps* *in Radopholus similis.* **a–k** Tissue localization of *Rs*-*cps* mRNA in *R. similis* using in situ hybridization. *Rs*-*cps* was expressed in the esophageal glands (**a**, **b**) and ovaries (**d**) of females. *Rs*-*cps* was expressed in the esophageal glands (**f**), testes and vas deferens (**h**) of males. *Rs*-*cps* was expressed in eggs (**j**). **c**, **e**, **g**, **i**, **k** no hybridization signal was observed in the control nematodes and eggs that were hybridized with a DIG-labeled sense *Rs*-*cps* RNA probe. *b* bursa; *e.g.* esophageal glands; *mb* medium bulb; *o* ovary; *s* stylet; *sp* spicules; *vd* vas deferens; *t* testis. **l** Expression of *Rs*-*cps* at different development stages in *R. similis*. The *bars* indicate the standard errors of the mean (n = 3), and *different letters* indicate significant differences (*p* < 0.05) between treatments

The tissue localization of *Rs*-*cpsin R. similis* was determined by in situ hybridization. *Rs*-*cps* mRNA was expressed in the esophageal glands and ovaries of females (Fig. 3a, b, d), the esophageal glands, testes and vas deferens of males (Fig. 3f, h), and the eggs of *R. similis* (Fig. 3j), as indicated by hybridization with a 478-bp DIG-labeled antisense mRNA probe. No hybridization signal was observed in nematodes and eggs when the control sense mRNA probe was used (Fig. 3c, e, g, i, k). The expression level of *Rs*-*cps* in *R. similis* at different development stages was detected using qPCR. The results showed that the *Rs*-*cps* mRNA transcript was present in all developmental stages of *R. similis* and that the highest transcript level was detected in females. The expression of *Rs*-*cps* in juveniles, males and eggs accounted for 64.4, 57.7 and 42.5 % of the expression level in females, respectively. *Rs*-*cps* expression was significantly lower in eggs than in juveniles and males (*p* < 0.05), but no significant difference was observed between juveniles and males (*p* > 0.05) (Fig. 3l).

### Effects of FITC and octopamine on *R. similis*

**Fig. 4:**
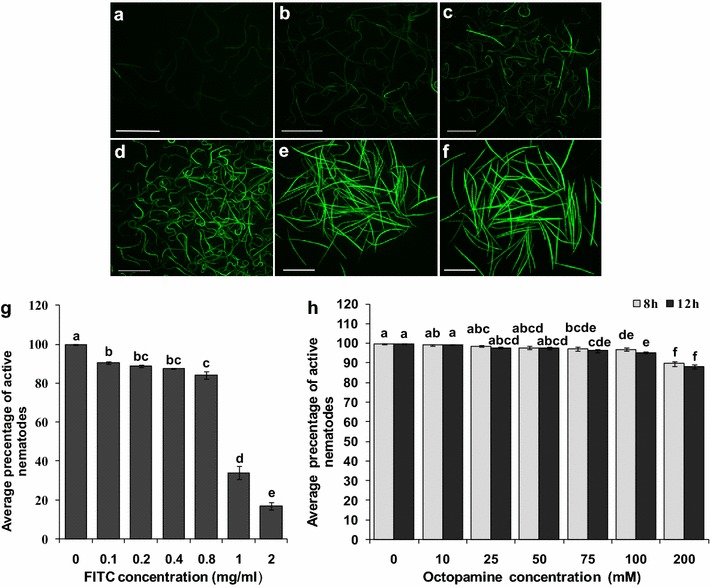
Effects of soaking mixed stages of *Radopholus similis* with FITC and octopamine. **a–f** FITC fluorescence in *R. similis* incubated for 8 h with different concentrations of FITC, as follows: **a** 0.1 mg/mL, **b** 0.2 mg/mL, **c** 0.4 mg/mL, **d** 0.8 mg/mL, **e** 1.0 mg/mL and **f** 2.0 mg/mL. The *scale bar* represents 500 μm. **g** Average percentages of active nematodes after worms were soaked with different concentrations of FITC for 8 h. **h** Average percentages of active nematodes after worms were soaked with different concentrations of octopamine for 8 and 12 h. The *bars* indicate the standard errors of the mean (n = 5), and *different letters* indicate significant differences (*p* < 0.05) between different treatments

The effects of FITC and octopamine on the activity of *R. similis* and the effect of octopamine on the uptake of dsRNA solution by nematodes were assessed using FITC as a visual marker. With the exception of the control group (without FITC), the nematodes in the soaking solutions with different concentrations of FITC all fluoresced after 8 h of incubation, and the fluorescence intensity in the nematodes increased as the concentration of FITC increased. Weak fluorescence was observed in nematodes soaked in solutions containing 0.1, 0.2 and 0.4 mg/mL FITC, whereas strong fluorescence was observed in nematodes soaked in solutions containing 0.8, 1.0 and 2.0 mg/mL FITC (Fig. 4a–f). In soaking solutions containing 1.0 and 2.0 mg/mL FITC, the percentage of inactive nematodes reached 66 and 83.2 %, respectively, significantly higher (*p* < 0.05) than the percentage in the control group (without FITC) and the groups incubated in the other four concentrations of FITC (0.1, 0.2, 0.4, 0.8 mg/mL). In soaking solutions containing 0.8 mg/mL FITC, the percentage of inactive nematodes was 15.8 %, significantly higher than the percentage in the solution containing 0.1 mg/mL FITC (*p* < 0.05). However, there was no significant difference (*p* > 0.05) in the percentage of inactive nematodes after the worms were incubated in solutions containing 0.1, 0.2 and 0.4 mg/mL FITC for 8 h (Fig. 4g). These results indicate that the FITC was toxic to *R. similis*. The toxic effect of FITC on nematodes was greater at 1.0 and 2.0 mg/mL than at the other four tested concentrations. There was no significant difference in the activity of *R. similis* when the nematodes were soaked in 0.2, 0.4 and 0.8 mg/mL FITC (*p* > 0.05). However, more nematodes displayed fluorescence at 0.8 mg/mL than at 0.2 and 0.4 mg/mL, and the fluorescent intensity was stronger. Therefore, 0.8 mg/mL FITC was used as a visual marker in the subsequent experiments.

After 8 h of incubation, there was no significant difference (*p* > 0.05) in the activity of *R. similis* in the groups soaked with octopamine at concentrations of 50 mM or less and the control group (without octopamine). Significant differences were observed in the activity of *R. similis* soaked with octopamine at concentrations greater than 50 mM and the control group (*p* < 0.05). However, activity of nematodes was reduced by only 10.2 % in the group soaked with 200 mM octopamine compared to the control group. When the incubation time was extended to 12 h, the activity of *R. similis* soaked in different concentrations of octopamine did not differ from that observed after incubation for 8 h (Fig. 4h). These results indicate that the detrimental effects of octopamine on the activity of *R. similis* are very small. Therefore, the ability of 50 mM octopamine to enhance the ingestion of the soaking solution by *R. similis* was assessed.

**Fig. 5:**
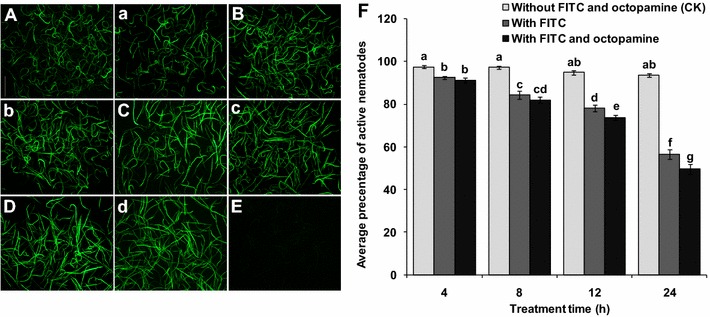
FITC fluorescence and viability in mixed-stages *Radopholus similis* incubated for different times in soaking solutions containing FITC and octopamine. **a–d** Expression in nematodes soaked in 0.8 mg/mL FITC for 4, 8, 12 and 24 h, respectively. **a–d** Expression in nematodes soaked in 0.8 mL/mg FITC and 50 mM octopamine for 4, 8, 12 and 24 h, respectively. **e** Nematodes soaked in water without FITC and octopamine were used as a control (CK). The *scale bar* represents 500 μm. **f** Viability of nematodes after soaking in FITC (0.8 mg/mL) and octopamine (50 mM) solution for 4, 8, 12 and 24 h. The *bars* indicate the standard errors of the mean (n = 5), and *different letters* indicate significant differences (*p* < 0.05) between treatments

When 0.8 mg/mL FITC was used as a marker, there was no obvious difference in fluorescence intensity between the mixed-stage *R. similis* that were soaked with 50 mM octopamine and those in soaking solution without octopamine after incubation for 4 h to 24 h (Fig. 5a–d). After incubation in FITC solution with or without octopamine for 24 h, the percentage of inactive nematodes reached 50.4 and 43.4 %, respectively, significantly higher (*p* < 0.05) than that observed in the control group (6.4 %) and the other incubation time groups (Fig. 5f). These results indicate that octopamine did not significantly enhance the ingestion of soaking solution by *R. similis* but instead had a detrimental effect on nematode activity. Therefore, we directly soaked *R. similis* in dsRNA soaking solution without octopamine in the subsequent RNAi experiments.

**Fig. 6:**
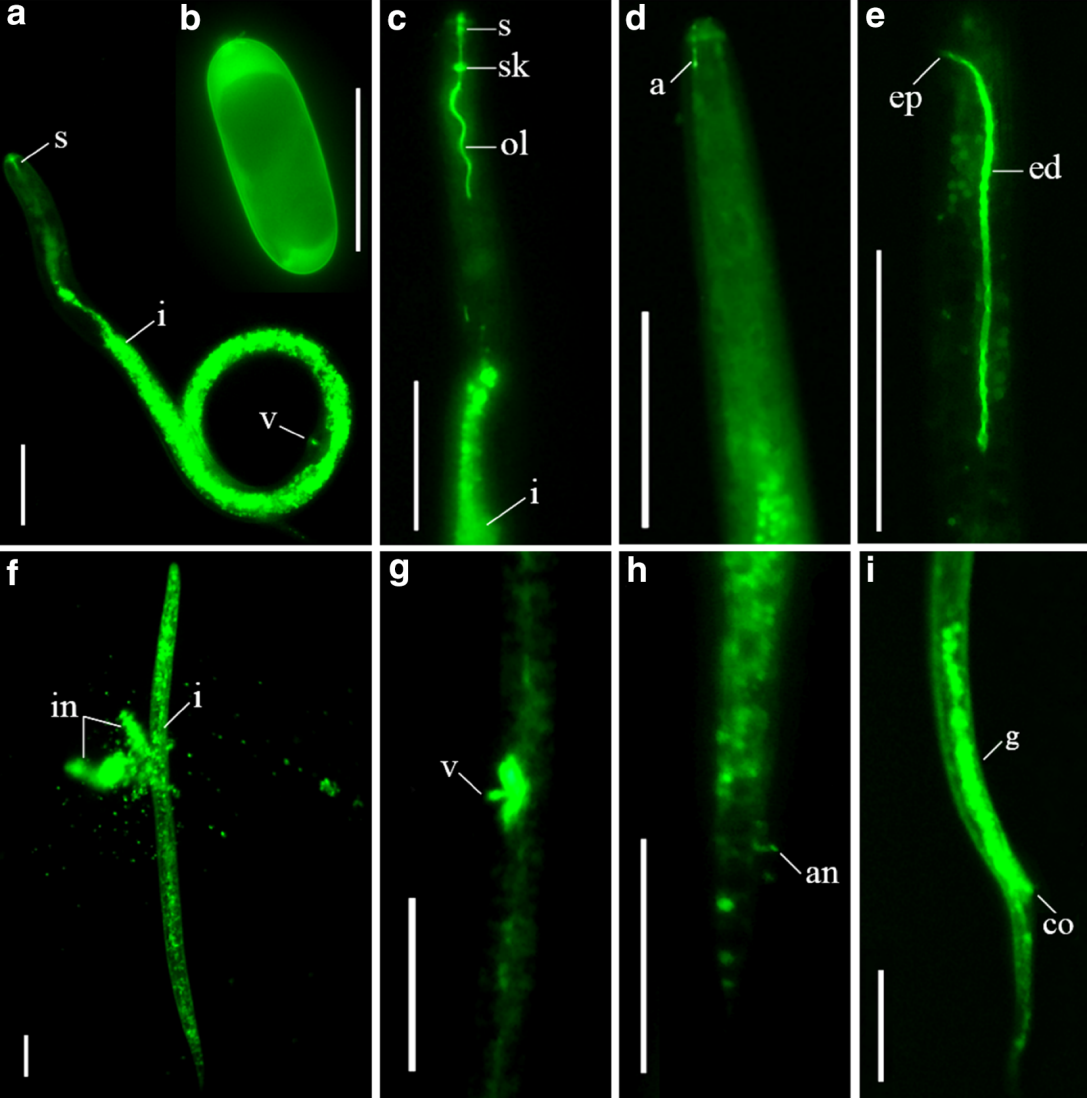
Fluorescence microscopy showing FITC and dsRNA uptake by *Radopholus similis* soaked in 0.8 mg/mL FITC for 8 h. **a**, **b** FITC fluorescence in the nematode and egg. **c** FITC fluorescence in the style, the esophageal lumen and the intestines in *R. similis*. **d** FITC fluorescence in the amphids. **e** A strong FITC signal was observed in the excretory ducts of *R. similis*. **f** A strong fluorescence signal was observed in the inclusion of the nematode body. **g** FITC fluorescence was observed in the vagina. **h** FITC entered the rectum via the anus. **i** FITC entered the reproductive system via the cloacal orifice. *a* amphids; *an* anus; *co* cloacal orifice; *ed* excretory duct; *ep* excretory pore; *g* gonads; *i* intestine; *in* inclusion of the nematode body, *ol* oesophageal lumen; *s* stylet; *sk* stylet knobs; *v* vagina. The *scale bar* represents 50 μm

After incubation for 8 h in dsRNA solution with FITC (0.8 mg/mL as a visible marker), strong fluorescence signals were observed in the stylet, esophageal lumen, intestine, amphids, excretory duct and anus of *R. similis* (Fig. 6c–e, h). Fluorescence microscopy also indicated that the soaking solution diffused into the body of *R. similis* via the vagina and cloacal orifice (Fig. 6g, i) and even the egg via the egg shell (Fig. 6a, b). Strong fluorescence signals were observed in these structures. Strong fluorescence signals were observed in the inclusions of the nematodes when the bodies of *R. similis* were pierced using an insect pin (Fig. 6f). Using FITC as a marker, this study is the first to demonstrate that a neurostimulant is not required to induce *R. similis* to ingest dsRNA in a soaking solution via many of its organs in both vermiform nematodes and egg shells.

### Detection of RNAi efficiency

**Fig. 7:**
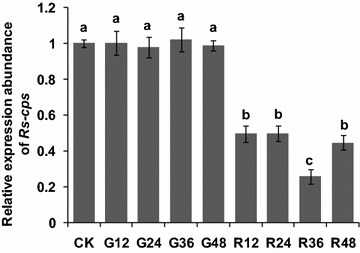
Expression of the *Rs*-*cps* mRNA in *Radopholus similis* treated with *Rs*-*cps* dsRNA for different times. CK, untreated nematodes; G12, G24, G36 and G48, expression in nematodes soaked in non-endogenous *egfp* dsRNA for 12 h, 24 h, 36 h and 48 h, respectively; R12, R24, R36 and R48, expression in nematodes soaked in *Rs*-*cps* dsRNA for 12 h, 24 h, 36 h and 48 h, respectively. The *bars* indicate the standard errors of the mean (n = 3), and *different letters* indicate significant differences (*p* < 0.05) between treatments

The silencing efficiency of *Rs*-*cps* in *R. similis* was determined by qPCR analysis of worms that were treated with *Rs*-*cps* dsRNA. The results showed that *Rs*-*cps* expression levels in nematodes decreased significantly (*p* < 0.05) by 50.5, 50.2, 74.4 and 55.4 % when the worms were treated with *Rs*-*cps* dsRNA for 12 h, 24 h, 36 and 48 h, respectively, compared to the expression level of *Rs*-*cps* in the untreated control nematodes (CK). *Rs*-*cps* expression was significantly lower (*p* < 0.05) in the nematodes treated with *Rs*-*cps* dsRNA for 36 h than in the other RNAi-treated groups. *Rs*-*cps* expression was significantly lower in the nematodes treated with *Rs*-*cps* dsRNA (*p* < 0.05) than in the CK group and the e*gfp* dsRNA-treated nematodes, but there was no significant difference (*p* > 0.05) between the latter two control groups (Fig. 7). Therefore, *Rs*-*cps* expression in *R. similis* was effectively inhibited by soaking the nematodes with *Rs*-*cps* dsRNA, and the silencing efficiency was highest at 36 h.

### Phenotype and reproduction of *R. similis* after RNAi

**Fig. 8:**
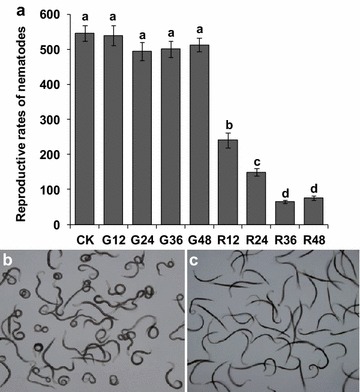
Phenotypes and reproduction of *Radopholus similis* after RNAi. **a** Reproductive rates of *R. similis* extracted from carrot disks at 50 days after inoculation with 30 females. CK: untreated nematodes; G12-G48: nematodes treated with non-endogenous *egfp* dsRNA for 12 h, 24 h, 36 h and 48 h, respectively; R12–R48: nematodes treated with *Rs*-*cps* dsRNA for 12 h, 24 h, 36 h and 48 h, respectively. The *bars* indicate the standard errors of the mean (n = 5) and *different letters* indicate significant differences (*p* < 0.05) between treatments. **b**, **c** The phenotypic observation of *R. similis*. Nematodes were soaked in *Rs*-*cps* dsRNA (**b**) and non-endogenous e*gfp* dsRNA solutions (**c**) for 36 h

After soaking the nematodes in *Rs*-*cps* dsRNA for 36 h, most of the *R. similis* became twisted and circled with trembling (Fig. 8b–c). The effect of *Rs*-*cps* silencing on the reproductive capabilities of *R. similis* was assessed by inoculating the nematodes on carrot disks. After culture on carrot disks for 50 d, nematodes treated with *Rs*-*cps* dsRNA for 12, 24, 36 and 48 h had a significantly lower reproductive rate (reproductive rate = final nematodes ⁄ initial nematodes) than untreated (CK) and e*gfp* dsRNA-treated nematodes (*p* < 0.05). Nematodes treated with *Rs*-*cps* dsRNA for 36 h had the lowest reproductive rate (65.1), but no significant difference (*p* > 0.05) was observed between the 36 and 48-h treatments. The reproductive rates of the nematodes treated with *Rs*-*cps* dsRNA for 36 and 48 h were significantly lower (*p* < 0.05) than those treated for 12 h (149.3) and 24 h (240.8), but no significant difference (*p* > 0.05) was observed between the latter two groups. The reproductive rates of the untreated and e*gfp* dsRNA-treated nematodes were higher than 490, and no significant difference (*p* > 0.05) was observed among the untreated and e*gfp* dsRNA-treated groups (Fig. 8a).

### The pathogenicity of *R. similis* decreases significantly after RNAi

**Fig. 9:**
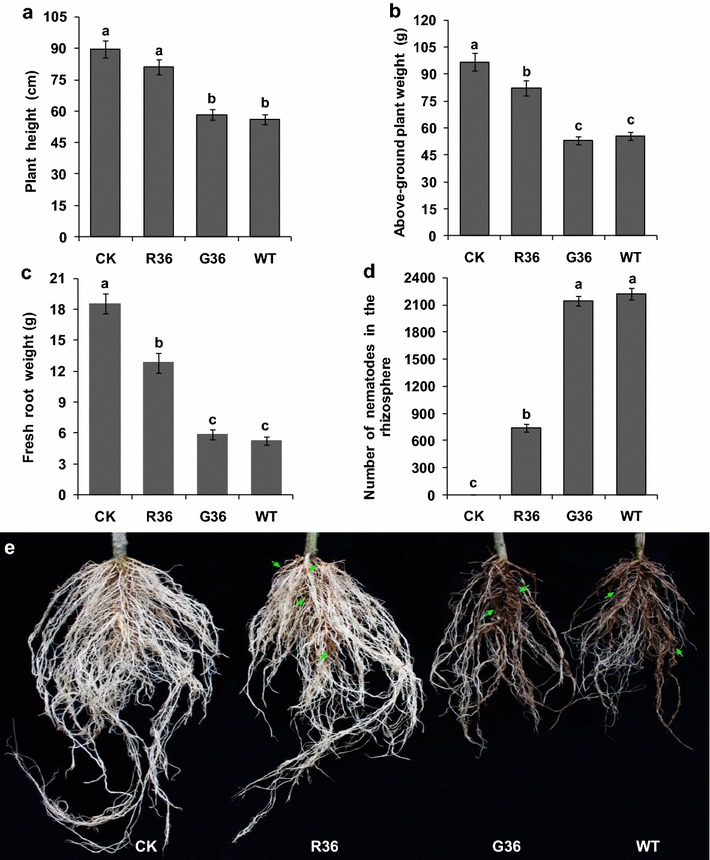
Effect of RNAi on the pathogenicity of *Radopholus similis*. Plant height (**a**), above-ground plant weight (**b**), fresh root weight (**c**), number of nematodes in the rhizosphere (**d**) and root infection symptoms (**e**) of tomato plants 2 months after inoculation with 1000 nematodes treated under different conditions. CK: uninoculated healthy tomato plants; R36: inoculated with nematodes treated with *Rs*-*cps* dsRNA for 36 h; G36: inoculated with nematodes treated with non-endogenous e*gfp* dsRNA for 36 h; WT: inoculated with untreated nematodes. The *bars* indicate the standard errors of the mean (n = 5), and *different letters* indicate significant differences (*p* < 0.05) between treatments

The effect of *Rs*-*cps* RNAi on the pathogenicity of *R. similis* was analyzed by inoculating nematodes on tomato plants under different treatment conditions. At 60 d after inoculation, the plant heights, fresh shoot weights and fresh root weights of the tomato plants inoculated with the nematodes treated with *Rs*-*cps* dsRNA for 36 h were 81.12 cm, 82.44 and 12.81 g, respectively, significantly higher (*p* < 0.05) than the plants inoculated with untreated nematodes (56.22 cm, 55.56 and 5.27 g, respectively) or e*gfp* dsRNA-treated nematodes (58.42 cm, 53.02 and 5.82 g, respectively) but lower than those of uninoculated healthy plants (89.52 cm, 96.74 and 18.55 g, respectively). There was a significant difference in fresh shoot weights and fresh root weights between uninoculated healthy tomato plants (CK) and the plants inoculated with nematodes treated with *Rs*-*cps* dsRNA for 36 h (*p* < 0.05), but no significant difference in plant height was observed between the two treatments (*p* > 0.05). There was no significant difference (*p* > 0.05) in the three growth parameters between tomato plants inoculated with e*gfp* dsRNA-treated and untreated nematodes (Fig. 9a–c). The number of nematodes in the rhizospheres of tomato plants inoculated with nematodes that were treated with *Rs*-*cps* dsRNA for 36 h was 742, significantly lower (*p* < 0.05) than that observed in the plants inoculated with untreated (2224) and e*gfp* dsRNA-treated nematodes (2148). The latter two treatments did not differ significantly (*p* > 0.05) (Fig. 9d). The degree of root damage was much lower in tomato plants inoculated with *Rs*-*cps* dsRNA-treated nematodes than in the control plants inoculated with untreated and e*gfp* dsRNA-treated nematodes, but spots and local rot caused by nematodes treated with *Rs*-*cps* dsRNA were observed on these plant roots. The root systems of the tomato plants inoculated with untreated and e*gfp* dsRNA-treated nematodes were significantly smaller and severely damaged compared to those of healthy plants, indicating obvious root rot (Fig. 9e). The inoculation tests thus demonstrated that the pathogenicity of *R. similis* was significantly decreased after treatment with *Rs*-*cps* dsRNA for 36 h, whereas the pathogenicity of *R. similis* was not impacted by treatment with non-specific e*gfp* dsRNA.

## Discussion

Cysteine proteinases play important biological roles in nematodes and many other animal parasites [9–12, 17]. In vitro RNAi-induced gene silencing has been successfully applied to free-living nematode *C.elegans* and the migratory endoparasitic plant-parasitic nematode *B. xylophilus*, without the addition of a neurostimulant [31, 32]. Delivery of dsRNA via ingestion is difficult in sedentary endoparasites such as cyst nematodes and root-knot nematodes because these second-stage juveniles (J2s) feed only following the establishment of a feeding site inside the root, and do not ingest substances prior to this stage [27]. However, uptake of dsRNA soaking solution has been induced in these nematode J2s by adding neurostimulant [27, 28, 44]. RNAi has been applied to *R. similis* by soaking the nematodes in dsRNA solution that does not contain neurostimulant [19, 20, 34]. The ability of the neurostimulant octopamine to enhance the ingestion of soaking solution by *R. similis* and the potential detrimental effects of octopamine on nematode activity have not been evaluated. Tan et al. [33] reported that the percentage of activity *Pratylenchus thornei* was reduced by only 12 % after the worms were incubated with 100 mM octopamine for 16 h compared to the control group. Tan et al. also demonstrated that more than 90 % of both *P. thornei* and *P. zeae* were dead after only 4 h of incubation in 1 % resorcinol (another neurostimulant) [33]. In this study, using FITC as a marker, we demonstrated that *R. similis* can ingest dsRNA soaking solution without stimulation by a neurostimulant, similar to *C. elegans* and *B. xylophilus* [27, 31, 32]. We also confirmed that the neurostimulant octopamine did not significantly enhance the ingestion of dsRNA soaking solution by *R. similis* but instead had a detrimental effect on nematode activity. In addition, the dsRNA soaking solution may diffuse into the body of *R. similis* not only via the esophageal lumen but also via the amphids, excretory duct, vagina, anus, cloacal orifice and egg shells.

The *cb* gene is mainly expressed in the intestines in *C. elegans* [45], *A. cantonensis* [46] and *Haemonchus contortus* [47] and in the cecal epithelial cells, digestive tract and reproductive system in *F. gigantic* [11]. Li et al. [20] reported that *Rs*-*cb*-*1* is expressed in the esophageal glands, intestines and gonads of females, the testes of males, and juveniles and eggs in *R. similis*. Hashmi et al. [10] reported that *Ce*-*cl*-*1* is widely expressed in the intestines, hypodermal cells and eggshells of *C. elegans*. Guiliano et al. [14] confirmed that *cl* is highly expressed in the esophageal glands of *B. malayi* and *B. pahangi* infective third-stage larvae. In the plant parasitic nematode *M. incognita*, *Mi*-*cl*-*1* is expressed in the intestines of young and mature female nematodes [30]. In this study, the expression and localization of *Rs*-*cps* in *R. similis* were associated with the biological functions of cathepsin. The expression of *Rs*-*cps* in the esophageal glands of *R. similis* may facilitate the disruption of plant defensive responses, the establishment of a parasitic relationship, and the rapid digestion of host cells to obtain the nutrients necessary for metabolism and other physiological functions. These findings are also consistent with the functions of esophageal secretions that have been documented in other plant parasitic nematodes. The secretions of esophageal glands produced by plant parasitic nematodes are thought to play key roles throughout the process of parasitism [48, 49]. *Rs*-*cps* was located in the eggs and reproductive system of *R. similis*, possibly because CPS plays important roles in development, reproduction and cell differentiation in *R. similis*. The females of *R. similis* are responsible for both infection and reproduction; *Rs*-*cps* expression is therefore highest in females. Thakur et al. [21] reported that the expression level of *Ha*-*cps* was also highest in females of *H. avenae*. *Rs*-*cps* expression was significantly higher in infective juveniles than in eggs, likely because the successful destruction of host defense responses and the establishment of a parasitic relationship are a precondition for other functions to be implemented by *R. similis*. In this study, *Rs*-*cps* expression was lowest in eggs, 42.5 % of the expression level in females. This result reveals that CPS may also play important roles in embryo formation and cell differentiation in *R. similis*. Previous studies have shown that *Rs*-*cb*-*1* plays vital roles in reproduction, development and pathogenesis in *R. similis* [19, 20]. B-, L- and S-like cathepsin belong to the cysteine protease family, and they share a close genetic relationship and similar structures. Therefore, these proteases may have similar biological functions during processes such as infection and pathogenesis in nematodes.

To further define the functions of *Rs*-*cps* and to explore the possibility of using this promising target for controlling *R. similis*, an RNAi experiment was performed in this study. Treatment with *Rs*-*cps* dsRNA significantly decreased the expression levels of *Rs*-*cps* and the reproductive rates of *R. similis* compared to the control groups. The silencing efficiency of *Rs*-*cps* was highest and the reproductive rate of *R. similis* was lowest on carrot disks after treatment with *Rs*-*cps* dsRNA for 36 h. In the subsequent pot experiments, the pathogenicity of *R. similis* to tomato plants was also significantly reduced after treatment with *Rs*-*cps* dsRNA for 36 h. These results are consistent with the tissue localization of *Rs*-*cps* in *R. similis*. Therefore, we confirmed that *Rs*-*cps* plays key roles in reproduction and pathogenesis in *R. similis* and that CPS might be a promising target for controlling this nematode. Here we first report the use of RNAi in studying the functions of *cps* in plant parasitic nematodes and suggests a promising new target for controlling *R. similis*. An RNAi effect could be generated in nematodes by feeding on transgenic plants expressing a specific target gene dsRNA [20, 27, 38]. Therefore, these findings support applications aimed at controlling plant parasitic nematodes by constructing *Rs*-*cps* plant RNAi vectors and obtaining transgenic plants that express specific hairpin dsRNAs of reproduction-, parasitism- and pathogenesis-related genes and merit further investigation.

## Conclusions

This is the first work to examine the functions of *cps* from *R. similis*. *Rs*-*cps* mRNA was expressed in the esophageal glands and ovaries of females, the esophageal glands, testes and vas deferens of males and the eggs of *R. similis*. *Rs*-*cps* was expressed at varying levels in all developmental stages of *R. similis*. The expression level of *Rs*-*cps* was significantly suppressed in nematodes and that the reproductive capability and pathogenicity of *R. similis* were significantly reduced after RNAi. These results indicated that *Rs*-*cps* plays important roles in the reproduction, parasitism and pathogenesis of *R. similis* and could be used as a promising target for controlling plant parasitic nematodes.
